# Supplementary material for: Severity Classification of Laboratory Animal Procedures in Two Belgian Academic Institutions
Source: Animals (Basel). 2023 Aug 10;13(16):2581. doi: 10.3390/ani13162581 (PMC10451636; doi:10.3390/ani13162581)
Supplement: Supplementary file 1 [file animals-13-02581-s001.zip › animals-2539114-supplementary.pdf]

Supplementary tables

Table S1: Severity classification of administration

Below threshold
Non-invasive administration of substances, e.g., through drinking water or food for mammals; environmental water for aquatics
**Mild**
Routes without anesthesia:<br>- Mammals: subcutaneous, intravenous, intramuscular, intradermal, topical skin/eye/mucosal application<br>o Specific for rodents: intraperitoneal, gavage, intragastric in pups, intradermal in tail base/hock/ear pinna<br>- Aquatics: dorsal lymph sac<br>- Chickens: oculonasal<br>Routes with anesthesia:<br>- Intranasal<br>- Intratracheal via intubation or tracheotomy<br>- Intravitreal/cameral<br>- Intracerebral in anesthetized neonatal mouse pups<br>- Uncomplicated intracardial injection<br>- Intra-articular injection<br>- Retro-orbital plexus
**Moderate**
- Footpad injection<br>- Hydrodynamic tail vein injection

Table S2: Severity classification of sampling

FLUIDS	TISSUES
**Below threshold**	

Non-invasive collection of body fluids without affecting normal housing conditions (for metabolic cages, etc., see Table S13) and not causing more stress than during daily care and inspection, e.g., non-invasive urine collection, collection of feces	- Tissue sampling when this also identifies the animal including:<br>- Ear biopsies also used for identification<br>- 3rd phalanx amputation (day 5-7) used for mouse/rat identification<br>- Non-invasive mucus sampling for genotyping in zebrafish
**Mild**	
- Blood sampling respecting following basic rules regarding volume:<br>o Max 10% of total body volume on any single occasion<br>o < 25% of total blood volume in 28 days for repeated sampling<br>o Through one of the following techniques<br>▪ Rodents: saphenous vein, tail vein, sublingual vein, mandibular vein, jugular vein with or without anesthesia<br>▪ Rabbits: marginal ear vein<br>▪ Other species: normal venous access routes like jugular vein, saphenous vein, etc.<br>o Catheterization: see Table S3<br>- Urine collection through cystocentesis or catheterization<br>- CSF sampling under anesthesia (max 10% of total CSF volume (±40 µl in a mouse, ± 400 µl in a rat)<br>- Retro-orbital bleeding under anesthesia	- Tissue sampling for genotyping when the latter is not used for identification, e.g., tail and ear biopsies in rodents, fin clipping in zebrafish<br>- Sperm and oocyte collection (under anesthesia) in zebrafish<br>- Oocyte collection in xenopus by gentle squeezing<br>- Other types of tissue sampling usually fall in the category 'minor surgery' and are discussed in Table S3
**Moderate**	
- Blood sampling of more than 'mild' volumes (see above) with fluid replacement	
**Severe**	
Blood sampling of more than 'mild' volumes through an approved method without fluid replacement	

Table S3: Severity classification of surgery and surgical induction of disease

Mild
- Subcutaneous implantation of<br>o Osmotic pump or transponder <5% of body weight<br>o Foreign material, e.g., bone construct, catheter fragment, meshes, hormone pellets<br>o Biological tissues, e.g., tracheal rings, pericardium, tumor fragments/cells, etc.<br>- Tooth extraction with appropriate analgesia<br>- Bleeding time test by tail tip amputation or a small incision in the tail<br>- Catheterization of superficial vessels (a. carotis, v. jugularis, v. auricularis) without opening of body cavities and no restraint afterwards<br>- Endoscopic procedures (via natural orifices)<br>- Surgical oocyte recovery in Xenopus<br>- Optical nerve crush in zebrafish
**Moderate**
- Major surgery with opening of body cavities and appropriate analgesia, e.g., thoracotomy, laparotomy (including in utero electroporation and implantation of, e.g., telemetry transmitters, osmotic pumps, etc.), laparoscopy, orchidectomy, caesarian section, craniotomy (including implantation of catheters in ventricles of the brain or electrodes in the brain if the animals retain their freedom of movement)<br>- Removal of 'non-vital' organs through laparotomy: ovariectomy, vasectomy, splenectomy, hysterectomy, hypophysectomy with hormonal substitution<br>- Surgery causing transient or moderate impairment of locomotion (animals still capable of accessing food and water), e.g., degenerative cervical myelopathy, spinal cord injury in mice P5-P10<br>- Major or minor surgery causing temporary loss of function of specific organs/senses/body systems or permanent loss of function that can only be detected using specific tests and does not negatively affect the animal in standard lab housing conditions, e.g., stroke only causing alterations in specific behavioral tests, eye enucleation in mice, bowel resection depending on location and extent, limited myocardial infarction, surgery causing osteoarthritis, nephrectomy < 50%, limited cranial impact<br>- Orthopedic surgery with effective stabilization and wound management<br>- Organ/cell transplantation or device testing where rejection/failure does not lead to severe distress or impairment of the general condition of the animal, e.g., implantation of islets/pituitary/thymic cells under the renal capsule, implantation of cardiac assist device (with preservation of functional heart), organ transplantation with effective management of rejection, organ transplantation wherein rejection does not lead to severe distress as function is taken over by the contralateral organ, e.g., lung transplantation in mice<br>- Subcutaneous implantation of large (>5% of body weight) pumps or transmitters<br>- Implantation of a headpost

- Implantation of an imaging window (dorsal skinfold, cranial, etc.)
**Severe**
- Major surgery without proper analgesia<br>- Surgery causing severe impairment of locomotion, e.g., creation of unstable fractures, parabiosis in mice, fetal sheep surgery causing spina bifida, attachments of implants on the locomotor system or other large implants that restrict movement, spinal cord injury in adult mice<br>- Major or minor surgery causing permanent or progressive loss of function of specific organs/senses/body systems which severely affects animals in standard lab housing conditions, e.g., nephrectomy > 50%, surgery causing progressive heart failure (e.g., aortic banding), large myocardial infarction, surgery causing permanent paresis/paralysis (e.g., stroke), severe cranial impact, bowel resection depending on location and extent, hypophysectomy without hormonal substitution<br>- Organ/cell transplantation or device testing where rejection/failure may lead to severe distress, death or impairment of the general condition of the animal, e.g., implantation of artificial heart (as replacement of functional heart), organ transplantation without effective management of rejection, implantation of cardiac valve, implantation of artificial lung<br>- Surgery (intentionally) causing severe infection or sepsis, e.g., cecal ligation and punction, surgical endocarditis model, implantation of infected material in bones and joint structures<br>- Induction of severe trauma<br>- Sternotomy

Table S4: Severity classification of clinical signs

	Sub-threshold	Mild	Moderate	Severe
MAMMALS				
Body weight				
**Body weight (BW) loss animals not in growth phase**		Up to 10% BW loss<br>10-15% BW loss over more than 7 days	10–15% BW loss within 7 days<br>15-20% BW loss over more than 3 days	15-20% BW loss within 3 days<br>>20% BW loss
**Body weight rodents < 10 days**			No weight gain in 24h	Weight loss in 24h
**Body weight growing animals/juvenile**		Growth stop up to 2 days<br>Reduced growth up to 2 weeks	Growth stop 3-7 days<br>Reduced growth > 2 weeks	Growth stop > 7 days<br>Weight loss

**(including rodents > 10 days)**				
**Body Condition Score (BCS) (3 = normal)**		BCS 4 (overcondition)	BCS 2 (undercondition) or 5 (obese)	BCS 1 (emaciated)
**Food consumption**	90-100% of normal consumption	90-60% of normal consumption lasting no more than 6 days	90-60% of normal consumption lasting more than 7 days<br>< 40% of normal consumption lasting max 3 days	< 40% of normal consumption lasting more than 3 days
**General clinical signs**				
**Piloerection**		<8h	8h–5 days	>5 days
**Hunched posture**		<8h	8h–5 days	>5 days
**Subdued behavior**		Subdued but responsive lasting no more than 24h	Subdued but responsive lasting more than 24h<br>Subdued even if provoked lasting no more than 2 days	Subdued even if provoked lasting more than 2 days<br>Unresponsive when provoked
**Stereotypic behavior**		Intermittent stereotypic behavior	Frequent stereotypic behavior not affecting general health	Stereotypic behavior that cannot be stopped impacting health and welfare (e.g., lesions, poor body condition)
**Trembling**		<8h	8h–5 days	>5 days
**Dehydration**		Dehydration of < 10% characterized by:<br>- Mildly sunken eyes (appear >75% open)<br>- Skin tent <2 seconds<br>- Tongue moist	Dehydration of 10-15% characterized by:<br>- Skin tent >2 seconds<br>- Sunken eyes (appear half closed)<br>- Tongue dry	Dehydration of >15% characterized by:<br>- Skin tent >5 seconds<br>- Completely closed or severely sunken eyes<br>- Tongue parched
**Abdominal distention**		Visual abdominal distention by gas or fluid not causing other clinical signs like pain, dyspnea, increase in BW, etc.	Marked abdominal distention not causing other clinical signs like pain and dyspnea but with abnormal BW increase	Severe abdominal distention causing a tense/painful abdomen and/or dyspnea<br>Abdominal hernia

**Skin discoloration**			Pale extremities	Jaundice, cyanosis, anemic
**Body temperature**			Fever/hypothermia responding to treatment	Fever/hypothermia not responding to treatment or intentionally left untreated.<br>Rodents feeling cold when touched.
**Vocalization**		Transient vocalization	Intermittent vocalization or when provoked	Distressed or unprovoked vocalization
**Mobility**				
**Abnormal gait—lameness**		<8h	8h–5 days	>5 days
**Paresis**			Paresis	
**Paralysis**			Paralysis of one or more limbs <24h when the animal is able to move around the enclosure and has the ability to feed itself when food is presented	Paralysis of one or more limbs <24h when coupled with signs such as marked weight loss, inappetence or changes in behavior such as aggression.<br>Paralysis of one or more limbs >24h
**Respiration**				
**Respiration**		Subtle change in rate or effort during activity but normal at rest, regular rhythm	Obvious change in rate or effort during activity and at rest<br>Occasional irregular rhythm<br>Small amount of nasal discharge or sneezing	Reduced rate (easy to count in rodents) at rest and when active. Irregular rhythm.<br>Appears to require effort (gasping, open mouth breathing)<br>Noisy breathing<br>Nasal discharge affecting breathing
**Neurologic**				

**Head tilt**		Mild or occasional head tilt	Persistent head tilt but able to eat/drink	Persistent head tilt causing problems eating/drinking
**Loss of coordination**		Occasional loss of coordination but can still move well	Altered gait (e.g., hopping, wobbling, circling, wide stance and weak) but able to navigate the cage and able to access food and water	Marked abnormal gait impairing ability to move and function normally causing difficulties in eating and drinking
**Seizures**			Occasional seizures of short duration and normal afterwards	Prolonged duration (minutes) and repetitive, possibly affecting ability to maintain weight or behavior (hyperactivity or aggressive) or seizures without complete loss of consciousness during seizure.<br>Status epilepticus (seizure lasting > 5 min or recurrent epileptic seizures which can only be stopped by medication)
**Other neurological signs**			Head nodding, facial or neck jerks, mouth chewing, rhythmic contractions/extensions of forelimbs and/or hind limbs with or without loss of balance	All neurological signs impairing ability to move and function normally.
**Eyes**				
		Microphthalmia, anophthalmia, cataracts (opacity of the lens) with no evidence of inflammation or discomfort	Evident yellow/white discharge around eyes<br>Squinting (50-75% open) and eyelids or conjunctiva inflamed (red, swollen)<br>Scratching eyelids but not causing lesions	Severe yellow/white discharge that stains fur around eyes or legs.<br>Enlarged or bulged eye.

		Mild discharge quickly groomed away<br>Mild squinting (>75% open)<br>Small pinpoint area of damage/change to cornea	Irregular corneal surface and/or cloudy (25% of cornea) with watery eyes	Squinting (<50% open) and inflamed eyelids or conjunctiva<br>Scratching eyelids resulting in lesions<br>Irregular corneal surface and cloudy (>25% of cornea) with watery eyes and squinting
**GI tract**				
**Vomiting**		Occasional vomiting, hydration and weight normal	Daily vomiting, hydration and weight normal	Frequent vomiting causing dehydration/weight loss
**Diarrhea**		Intermittent soft feces but formed or daily paste-like feces, hydration normal	Daily watery diarrhea with occasional formed soft feces<br>Small area of fecal staining around anus. hydration normal	Only watery or bloody diarrhea resulting in dehydration<br>Large area of fecal staining on fur or visible fecal smearing on cage wall.
**Skin**				
**Prolapses**		Minimal exposure of tissue (<1 mm)<br>Intermittent prolapse but tissue healthy<br>Tissue can easily be reduced<br>Animal can urinate and defecate	Moderate amount of tissue exposed which requires treatment to reduce<br>Tissue healthy and animal can urinate and defecate	Fully prolapsed tissue.<br>Tissue not healthy (red, swollen, bleeding, dry, necrotic, etc.). Requires treatment to reduce and improve tissue health.<br>Unsure if animal can urinate and defecate
**Skin wounds**		Size:<br>- Mice: <3 mm body, <1mm face<br>- Rats: <5 mm body, <3 mm face<br>Skin red, mild hair loss	Size:<br>- Mice: 3-10 mm body, 1-3 mm face<br>- Rats: 5-30 mm body, 3-10 mm face	Size:<br>- Mice: >10 mm body, >3 mm face<br>- Rats: >30 mm body, >10 mm face

			Skin red, swollen, hair loss, some damage but dry or scabbed, possible scratching	Skin open with signs of infection (wet discharge of blood or pus) or open to muscle or bone.<br>Evidence of severe self-mutilation.
**Scratching**		Occasional scratching	Frequent scratching, still periods of not scratching, stops when disturbed	Constant scratching (even when disturbed)
**Others**				
**Urination**			Mild urine scalding (wet, reddish skin).<br>Polyuria w/o dehydration	Large area of urine scalding (wet, reddish skin)<br>Inability to urinate<br>Polyuria with dehydration
**AQUATICS**				
**Zebrafish larvae up to 12 days post fertilization**				
**Overall morphology:**<br>**- Body axis:** body length, spine curvature, tail/somite formation<br>**- Developmental rate:** pigmentation, time of hatching, yolk sac<br>**- Craniofacial development:** size of head/eyes, formation of craniofacial structures	Body axis, developmental rate and craniofacial development are indistinguishable compared to those of wild type fish	Body axis and/or developmental rate and/or craniofacial structures are subtly different	Body axis and/or developmental rate and/or craniofacial structures are obviously different	Body length is strongly reduced and/or the spine is strongly curved and/or the tail is not detached and/or the number of somites is strongly reduced<br>Developmental rate is strongly delayed<br>Size of the head is strongly reduced and/or the size of the eyes is strongly reduced and/or almost complete loss of the craniofacial structure

**Necrosis**	No indication of necrotic tissue	Necrosis <30% and does not seem to be organ specific	Total necrosis (overall and organ specific) 30-50%	Total necrosis (overall and organ specific) >50%
**Swim bladder—development and size**	Indistinguishable compared to those of wild type fish	Developmental rate is subtly delayed and/or inflation is <50% reduced	Developmental rate is obviously delayed and/or inflation is >50% reduced	Almost complete loss of the swim bladder
**Posture (i.e., roll and pitch axis and buoyancy control)**	Roll- and pitch-axis and the buoyancy control are indistinguishable compared to those of wild type fish	Occasional and/or subtle loss of the posture<br>Position in the water is normal or only subtly different	Frequent and/or obvious loss of posture<br>Position in the water column is obviously changed	Almost complete loss of posture<br>Almost complete loss of buoyancy control
**Cardiac function (i.e., heart rate and rhythm and heart area/pericardial edema)**	Heart rate and rhythm are indistinguishable compared to those of wild type fish and no indication of pericardial edema	Heart rate is subtly different but the heart rhythm is regular<br>Heart area is increased with <50%	Heart rate is obviously different<br>Heart rhythm is occasionally irregular<br>Heart area is increased with >50%	Heart rate is strongly reduced or erratic<br>Heart rhythm is frequently irregular<br>Heart area is increased with >100%
**Touch response (i.e., latency time, speed**, **swimming characteristics and travel distance**	Touch response is indistinguishable compared to those of wild type fish	Touch response is subtly reduced and/or different	Touch response is obviously reduced and/or different	Almost complete loss of the touch response
**Adult zebrafish (sexual mature)**				
**BCS**	BCS3	BCS2 (undercondition), BCS4 (overcondition)	BCS5 (obese)	BCS1 (emaciated)
**Abdominal distention**	Distention of the abdomen is normal	Mild but symmetric swelling (e.g., due to edema or egg bounding)	Moderate but symmetric swelling	Severe but symmetric swelling; asymmetric swelling (e.g., due to tumor or other abnormal masses)
**Size and spinal development**	Growth rate (size) is normal and spinal	Minor reduction in size; curvature/kink with no impact	Obvious reduction in growth rate; curvature/kink with only a mild/moderate	Spinal deformities (e.g., scoliosis) that make natural

	deformities (e.g., presence of curvature/kink) are absent	on natural behavior (e.g., swimming, feeding, breeding, …)	impact on natural behavior (e.g., swimming, feeding, breeding, …)	behavior (e.g., swimming, feeding, breeding, …) impossible
**Head and eye**	Physiologic appearance is normal	Underdeveloped upper and lower jaw; one eye is blind and/or damaged (e.g., cloudy, bloody or discolored) and/or sunken	Both eyes are damaged but with only a mild impact on natural behavior (fish can still feed); minor exophthalmos	Abnormal head size and/or tumor of the head or eye and/or fully blind that make natural behavior (such as feeding) impossible; exophthalmos or sunken eyes
**Skin and scales**	General appearance, skin pigmentation (not due to GA), condition of scales and mucus production are normal	Pigmentation: subtle patchy loss or increase; change in pattern<br><br>Scales: very limited loss/damage of scales without bleedings or signs of infections.<br>Appearance: small superficial lesion (e.g., wound, ulcer, abscess or tumor) without bleeding or sign of infections and without affecting natural behavior	Pigmentation: obvious patchy loss or increase<br><br>Scales: loss/damage of scales without bleedings or signs of infections; scales are subtly raised<br>Appearance: Lesions with minor bleedings or signs of infection and/or limited covering with abnormal nodules but without affecting natural behavior; subtle difference in mucus production	Pigmentation: white/grey color<br>Scales: obvious loss/damage of scales; bleedings and/or sings of infections are present; scales are obviously raised<br>Appearance: several small lesions or single large superficial lesions; open lesion with bleeding and/or infection; lesions affecting natural behavior; abnormal production of mucus; covering with abnormal nodules (e.g., warts, cotton-like growths); presence of gas bubbles; presence of necrotic tissue
**Fins**	All fins are present and appear normal	Deformed, clamped or partial missing fin(s) without affecting	Deformed, clamped or partial missing fin(s) with a subtle effect on natural	Tail (caudal) or pectoral fin is missing; decayed fins with bleeding and/or infection

		natural behavior and without bleedings or signs of infection	behavior; decayed fins but without bleedings or signs of infection	and/or clear effect natural behavior; fin tumor
**Status of gill and operculum and breathing**	Gills do not show any lesions (e.g., thickened, pale, bleedings); opercula are normal developed/formed and the frequency of the opercular movements (breathing) is normal	Subtle gill lesions, protruding, missing or deformed operculum on one side; slightly increased or decreased opercular movements without additional signs of illness; gasping for air at the surface or water supply	Both opercula are deformed; distinctly increased or decreased opercular movements without additional signs of illness	Pale gills, loss of gill filaments and obvious bleeding; both opercula are missing and/or protruding; swollen gills<br><br>Distinctly increased or decreased opercular movements with additional signs of illness; erratic opercular movements or opercular movements are almost absent
**Swimming behavior**	Normal, calm and steady; occupying the entire water column	Slow; occasional loss of equilibrium: occasionally swimming up or downwards to regulate buoyancy	Frequent loss of equilibrium; continuously swimming at slight angle; frequently swimming up or downwards to regulate buoyancy; mainly at the surface or the bottom of the tank but still showing swimming behavior	Complete loss of equilibrium; sinking when not actively swimming; lying on the bottom or floating at the water surface
**Social interaction and activity**	Active but with periods of resting; schooling is visible; normal response to visual/tactile stimuli	Aggression and/or chasing; overly active and/or agitated and/or hypoactive signs; subtle escape and/or hiding and/or isolation behavior; subtle reduction in response to visual/tactile stimuli	Isolation from the school; obvious reduction in response to visual/tactile stimuli; rubbing against the tank surface	Almost complete loss of reaction to visual/tactile stimuli; uncontrolled, erratic swimming behavior (e.g., corkscrew, jumping, freezing)
**Food intake**	Foraging behavior and food intake are normal	Reduction in foraging behavior and/or food intake <24h	Reduction in foraging behavior <48h; total lack of feeding <24h	Reduction in foraging behavior 1-3 days, total lack of feeding <48h; prolapse of anus or intestines

Table S5: Severity classification of chemical disease models

	Sub-threshold	Mild	Moderate	Severe
**Heart and Circulation**				
**Atherosclerosis**	Causing atherosclerotic plaques by feeding high cholesterol diet not causing clinical signs under normal conditions	Causing atherosclerotic plaques by feeding high cholesterol diet causing no more than mild clinical signs (see Table S4)	Causing atherosclerotic plaques by feeding high cholesterol diet causing no more than moderate clinical signs (see Table S4)	Causing atherosclerotic plaques by feeding high cholesterol diet causing severe clinical signs (see Table S4)
**Hypertension**		Rodent hypertension causing no more than mild comorbidities as defined in clinical signs (see Table S4)	Rodent hypertension causing no more than moderate comorbidities as defined in clinical signs (see Table S4)	
**Thrombotic thrombocytopenic purpura**			Thrombotic thrombocytopenic purpura mouse model causing no more than moderate clinical signs (see Table S4)	Thrombotic thrombocytopenic purpura mouse model causing severe clinical signs (see Table S4)
**Neurology and sensory organs**				
**Paralysis**			Paralysis of one or more limbs <24h when the animal is able to move around the enclosure and has the ability to feed itself when food is presented, e.g., early stages of experimental autoimmune encephalomyelitis in mice.	Paralysis of one or more limbs <24h when coupled with signs such as marked weight loss, inappetence or changes in behavior such as aggression. Paralysis of one or more limbs >24h, e.g., experimental autoimmune encephalomyelitis in mice.
**Seizures**		Epilepsy models causing focal periodic seizures and/or mild clinical signs (see table S4)	Epilepsy models:<br>- Causing occasional seizures of short	Epilepsy models causing:<br>- Repetitive seizures of prolonged duration

			duration and normal afterwards<br>- And/or moderate clinical signs (see table S4)	(minutes), possibly affecting ability to maintain weight or normal behavior (hyperactivity or aggressive)<br>- Seizures w/o complete loss of consciousness during seizure<br>- Status epilepticus (seizure lasting > 5 min or recurrent epileptic seizures which can only be stopped by medication)<br>- Severe clinical signs (see Table S4) or mortality, e.g., kanaic acid and pentylenetetrazole, ethyl ketopentenoate in zebrafish
**Endocrine, nutritional and metabolic**				
**Diabetes**		Rodent models of diabetes causing mild clinical signs as defined in clinical signs (see Table S4)	Rodent models of diabetes causing moderate clinical signs as defined in clinical signs (see Table S4)	Rodent models of diabetes causing severe clinical signs as defined in clinical signs (see Table S4)
**Respiratory system**				
**Emphysema**		Cigarette smoke inhalation model of chronic obstructive	Cigarette smoke inhalation model of COPD in mice with restraint (restrained 2	

		pulmonary disease (COPD) in mice without restraint	times/day, 5 times/week for several weeks)	
		Inhalation of diesel particles (low dosage, limited number of instillations)	Inhalation of diesel particles (high dosage, high number of instillations)	
		Elastase model of emphysema causing no more than mild clinical signs (see Table S4)	Elastase model of emphysema causing no more than moderate clinical signs (see Table S4)	Elastase model of emphysema causing severe clinical signs (see Table S4)
**Asthma**		Protein (ovalbumin/house dust mite) mouse asthma model<br>Chemical mouse asthma model		
**<u>Pulmonary fibrosis</u>**		Silica model of fibrosis (low dosage, limited number of instillations)	Silica model of fibrosis (high dosage, high number of instillations)	Bleomycin model of pulmonary fibrosis
**Acute Respiratory Distress Syndrome (ARDS)**				Models causing acute respiratory distress syndrome (ARDS) (e.g., LPS)
**GI tract**				
**Colitis**		Dextrane sulfate sodium (DSS)/TNBS (2,4,6-trinitrobenzenesulfonic acid) /Oxazolon mouse model of colitis causing no more than mild clinical signs (see Table S4)	DSS/TNBS/Oxazolon mouse model of colitis causing no more than moderate clinical signs (see Table S4)	DSS/TNBS/Oxazolon mouse model of colitis causing severe clinical signs (see Table S4)
**<u>Liver fibrosis</u>**			Carbon tetrachloride (CCl4) mouse model causing no more than moderate clinical signs (see Table S4)	CCl4 mouse model causing no more than severe clinical signs (see Table S4)

**Skin**				
**Psoriasis**		Imiquimob/Aldara psoriasis mouse model causing no more than mild clinical signs (see Table S4)	Imiquimob/Aldara psoriasis mouse model causing no more than moderate clinical signs (see Table S4)	Imiquimob/Aldara psoriasis mouse model causing severe clinical signs (see Table S4)
**<u>Itch</u>**		Histamine-cheek model of itch in the mouse		
**Locomotor system**				
**Arthritis**		Intra-articular papain arthritis model	Intra-articular collagenase arthritis model	Collagen-induced arthritis (CIA) and collagen antibody-induced arthritis (CAIA) rodent models of rheumatoid arthritis
**Urogenital**				
**Cystitis**				<u>Cyclophosphamide-induced cystitis model in rodents</u>
**Immune system**				
**Leukemia**	See Table S6			
**Graft versus Host Disease (GvHD)**		GvHD causing no more than mild clinical signs (see Table S4)	GvHD causing no more than moderate clinical signs (see Table S4)	GvHD causing severe clinical signs (see Table S4)
**Immunization**			Subcutaneous immunization with Complete Freund's Adjuvants	
**Others**	For immunological diseases giving clinical signs in 1 particular body system, see the respective body system			
**Toxicity tests and dose ranging studies**				
**Age**	Toxicity tests in animals not considered			

	laboratory animals, e.g., zebrafish larvae < 120 hours post fertilization (hpf)			
**Predictability**				Toxicity tests wherein it cannot be predicted that animals will adapt to the challenge without severe effect or mortality
**Clinical signs**		Tests wherein the humane endpoints applied correspond to mild clinical signs (see Table S4)<br>Tests wherein the predicted effects correspond to mild clinical signs (see Table S4)	Tests wherein the humane endpoints applied correspond to moderate clinical signs (see Table S4)<br>Tests wherein the predicted effects correspond to moderate clinical signs (see Table S4)	Tests wherein the humane endpoints applied correspond to severe clinical signs (see Table S4)<br>Tests wherein the predicted effects correspond to severe clinical signs (see Table S4)
**Toxicity tests leading to (possible) death**				Tests wherein:<br>-The compound in question can cause death and the study cannot be controlled to avoid mortality<br>-Where death is the endpoint and humane endpoints are/cannot be applied (i.e., survival curves)[#]<br>-Fatalities are to be expected and severe pathophysiological states are induced

Table S6: Severity classification of infectious diseases

<table>
<tr><th>Below threshold</th><th>Mild</th><th>Moderate</th><th>Severe</th></tr>
<tr><td colspan="4">Viral diseases</td></tr>
<tr><td>Viral infections causing no clinical signs and animals did not undergo a procedure to get infected or animals are infected in a stage before being regulated such as norovirus infection in zebrafish larvae (3 days post fertilization).</td><td>Viral infections causing no or mild clinical signs (see Table S4) and animals underwent a (mild) procedure to get infected such as:
- Mice: norovirus in C57Bl6, Japanese encephalitis vaccine strain in immunocompetent mice, vesicular stomatitis virus pseudotyped with Ebolavirus glycoprotein in C57Bl6, Zika virus in Swiss mice, respiratory syncytial virus, mouse norovirus in immunocompetent mice, Sars-CoV2 in most strains
- Rats: Rat hepatitis E virus in nude rats,
- Hamsters: vesicular stomatitis virus pseudotyped with Ebolavirus glycoprotein
- Ducklings: Duck hepatitis B</td><td>Viral infections causing moderate clinical signs or long-lasting mild clinical signs (see Table S4) such as:
- Hamster: Sars-CoV2

Viral infections causing no or mild clinical signs but animals underwent a moderate procedure to get infected such as through hydrodynamic tail vein injection (hepatitis B in mice) and footpad injection (chikungunya in mice)</td><td>Viral infections causing severe clinical signs, long-lasting moderate clinical signs (see Table S4) or infections causing lethality such as:
- Mice: mouse noro virus in AG129 mice, yellow fever, vesicular stomatitis virus pseudotyped with Ebolavirus glycoprotein in IFNAR−/− mice, Zika in AG129 mice, dengue in AG129 mice, Japanese encephalitis in AG129 mice, Sars-CoV2 in Ace2 mice
- Hamster: yellow fever, vesicular stomatitis virus pseudotyped with Ebola or Marburg virus</td></tr>
<tr><td colspan="4">Bacterial and fungal diseases</td></tr>
<tr><td></td><td>Bacterial or fungal infections causing no or mild clinical signs (see Table S4) and animals underwent a mild procedure to get infected such as:
- Mice: Aspergillus infection in immunocompetent mice, Cryptococcus neoformans
- Mice and rats: Candida biofilm studies in infected catheter fragments implanted SC</td><td>Bacterial or fungal infections causing moderate clinical signs or long-lasting mild clinical signs (see Table S4) such as:
- Mice: Escherichia coli cystitis model, Pseudomonas aeruginosa and Acinetobacter baumannii lung infection model, Staphylococcus</td><td>Bacterial or fungal infections causing severe clinical signs, long-lasting moderate clinical signs (see Table S4) or infections causing lethality such as:
- Mice: E. coli and S. aureus sepsis model, aspergillus in immunodeficient mice, systemic Candida albicans infection, Cryptococcus neoformans neurological model (IV)</td></tr>
</table>

		aureus muscle infection model	
**Parasitic diseases**			
	Parasitic infections causing no or mild clinical signs (see Table S4) and animals underwent a mild procedure to get infected or animals are killed before the onset of clinical signs such as African Trypanosomiasis in mice before onset of anemia and/or body weight gain due to hepatosplenomegaly	Parasitic infections causing moderate clinical signs or long-lasting mild clinical signs (see Table S4)	Parasitic infections causing severe clinical signs, long-lasting moderate clinical signs (see Table S4) or infections causing lethality such as malaria ARDS model in mice, African Trypanosomiasis in mice
**Combination models**			
			Combination of infections causing severe clinical signs, long-lasting moderate clinical signs (see Table S4) or infections causing lethality such as influenza-associated aspergillosiss

Table S7: Severity classification of neoplasm

	Mild	Moderate	Severe
**Body weight (BW) loss and Body Condition Score (BCS)**	BW loss up to 10%<br>BW loss of 10-15% loss ≤ 7 days	BW loss of 10-15% loss > 7 days<br>BW loss of 15-20% loss ≤ 2 days<br>Animals with BCS 2	BW loss of 15-20% loss ≤ 2 days<br>BW loss of >20% loss<br>Animals with BCS 1<br>All models of cancer cachexia
**Neurological signs**	Animals with circling/head tilt/ataxia not preventing them to eat/drink	Animals killed as soon they develop seizures, paresis/paralysis or ataxia/headtilt/circling preventing animals to eat/drink	
**Ascites, icterus, anemia**		Animals killed as soon they develop ascites, icterus or anemia	All models requiring ascitic tap

**Gastro-intestinal signs**		Animals with diarrhea without dehydration, rectal prolapse, bloody stool or constipation	
**Dehydration**			Animals remaining dehydrated despite treatment
**Respiratory signs**		Animals with tachypnea	Animals with dyspnea, cyanosis or positive stress test
**Locomotor signs**		Animals showing lameness	
**Urinary signs**		Animals with urinary retention, hematuria or polyuria	
**Pain**			Animals with pain despite analgesia<br>Models of cancer pain<br>Bone tumor models
**Moribundity/mortality**			Moribund animals or animals found dead
**Tumor characteristics**	Uncomplicated SC, mammary or skin tumor ≤ 2 cm^3	Ulcerated SC, mammary or skin tumor<br>Other tumors up to maximum size:<br>- Lung: max 50% of total lung volume<br>- Bone and muscle: max 10 mm diameter (± 500 mm^3).<br>- Brain: max 25% of the total volume (± 500 mm^3 depending on the strain).<br>- Abdominal tumors: max 1.5 cm^3<br>- Luminal occlusion of 50% or more; inability to pass food/feces.	

Table S8: Severity classification of other disease models

Sub-threshold	Mild	Moderate	Severe
		**Laser**	
	Laser-induced neovascularization model		
		**Hypoxia and hyperoxia**	
Control animals of bronchopulmonary dysplasia model in neonatal mice (housed in normoxia with mother)	Control animals of bronchopulmonary dysplasia model in neonatal rabbits (housed in normoxia but without mother)	Hypoxia-induced pulmonary hypertension mouse model	Bronchopulmonary dysplasia model in neonatal rabbits and mice (housed in hyperoxia)
		**Mechanical**	
		Mechanical overload arthritis model	Hindlimb immobilization in mice through suspension or 'casting'
		**Irradiation ± bone marrow transplantation**	
	Irradiation causing mild clinical signs (see Table S4)	Irradiation with a lethal dose with reconstitution of the immune system.<br>Irradiation with a sublethal dose with reconstitution without development of GvHD.<br>Irradiation causing moderate clinical signs (see Table S4)	Irradiation with a lethal dose without reconstitution of the immune system.<br>Irradiation with a (sub)lethal dose with reconstitution with development of GvHD.<br>Irradiation causing severe clinical signs (see Table S4)
		**Exposure to cold**	
Progressive exposure to cold to simulate hibernation in hamsters			

Table S9: Severity classification of abnormal housing and nutrition

	Mild	Moderate	Severe
**Abnormal housing for social species**			
**Solitary housing with sensory deprivation**	<24h	1-5 days	More than 5 days
**Solitary housing without sensory deprivation (visual, olfactory or auditory contact with conspecifics or equal)**	Always stays in mild		
**Metabolic cage**	<24h	1-5 days	More than 5 days
**Enclosure smaller than legal requirement but animal able to turn and walk**	Always stays in mild		
**Enclosure smaller than legal requirement and animal not able to turn/walk**	<24h	1-5 days	More than 5 days
**Grid floor**	<24h	1-5 days	More than 5 days
**Head fixation**		Head fixation during behavioral task/neurophysiology	
**Abnormal housing conditions intended to cause a specific disease/condition**	See Table S8		
**Food and water restriction (experimental condition, not anorexia)**			
**Food restriction**	Up to 5% BW loss (adults only)	5-20% BW loss (adults only)	
**Water restriction on dry food only**	Up to 12h	12-24h	More than 24h
**Water restriction on diet containing fluids (e.g., fruits)**	Up to 24h	More than 24h without clinical signs of dehydration (see Table S4)	More than 24h with clinical signs of dehydration (see Table S4)
**Modified diet**			
**Modified diet**	Modified diet not meeting nutritional requirements expected to cause mild clinical signs (see Table S4)	Modified diet not meeting nutritional requirements expected to cause moderate clinical signs (see Table S4), e.g., diet causing arteriosclerosis	Modified diet not meeting nutritional requirements expected to cause severe clinical signs (see Table S4), e.g., diet causing

		or obesity with moderate clinical signs (see Table S4) without spontaneous death	arteriosclerosis or obesity with spontaneous death

Table S10: Severity classification of behavioral tests

Mild
- Tests requiring (brief) handling of the animals in an unfamiliar environment:<br>o Barnes maze<br>o Elevated plus maze<br>o Marble burying test<br>o Novel object recognition<br>o Odor detection test<br>o Radial arm maze<br>o Social dominance test<br>o Social interaction<br>o Social preference/Social novelty<br>o T maze<br>o Y maze<br>o Morris water maze<br>o Dark–light box<br>o Open field exploration<br>- Tests requiring no more than 1 electrical shock, with a low current (max. 0.5mA for mice; max. 0.8mA for rats) and short duration (max. 2s):<br>o Passive avoidance<br>o Active avoidance<br>- Tests causing mild sleep deprivation (up to 6h)
**Moderate**
- Tests requiring repeated non-avoidable electrical shocks, with a low current (max. 0.5mA for mice; max. 0.8mA for rats) and short duration (max. 2s):<br>o Contextual fear conditioning

o Cue fear conditioning<br>o Treadmill with electrical shock<br>- Tests requiring moderate changes in housing (see Table S19):<br>o Conditioned place preference<br>o Prepulse Inhibition<br>o Startle<br>o Touchscreen<br>- Tests measuring despair:<br>o Forced swim test<br>- Tests causing moderate sleep deprivation (6 to 24h)
**Severe**
- Combination of different moderate testing protocols or moderate testing protocols lasting several weeks:<br>o Skinner boxes/operant condition<br>o Vogel water lick suppression<br>- Tests producing malaise:<br>o Conditioned taste aversion<br>- Forced swim or exercise tests with exhaustion as the end point<br>- Tests causing severe sleep deprivation (>24h)

Table S11: Severity classification of function measurements

**Non-recovery**
All function measurements performed entirely under anesthesia from which the animal does not recover, e.g., lung function measurement (with Flexivent®), terminal cystometry with urethane anesthesia, bronchoalveolar lavage, in vivo tibial loading.
**Below threshold**
Non-invasive measurement of body functions without disturbance of the animal, e.g., glucose measurement in urine or food/water intake measurement - In case these measurements are performed under abnormal housing conditions, see abnormal housing and nutrition (see Table S9)
**Mild**
- Motor tests requiring (brief) handling of the animals in an unfamiliar environment, e.g., Balance beam, Catwalk®, Cylinder test, DigiGAIT®, Grip strength, Rotarod, tracking locomotor behavior in zebrafish

- Non-invasive measurement of body functions performed under anesthesia or requiring short restraint (<1h), e.g., plethysmography, functional ultrasound, grip strength, electroretinogram, intraocular pressure measurement, electrocardiogram (ECG), non-invasive blood pressure measurement, local field potential recording in zebrafish, optical coherence tomography (OCT), electroencephalogram (EEG)<br>- Invasive function measurement performed under anesthesia or requiring short restraint (<1h), e.g., electromyography, invasive blood pressure measurement, nerve conduction velocity measurement, anal pressure measurement<br>- Function measurements requiring fasting causing mild weight loss as defined in clinical signs (See Table S4), e.g., fasting glucose measurement, gastric emptying through CO2<br>- Function measurement requiring minor surgery (see Table S3), e.g., function measurement requiring implantation of a subcutaneous implant<br>- Function measurements requiring food/water deprivation within mild limits (see Table S9)
**Moderate**
- Function measurement requiring major surgery (see Table S3), e.g., electrophysiology measurements requiring craniotomy, cystometry requiring laparotomy<br>- Function measurements requiring food/water deprivation within moderate limits (see Table S9)<br>- Non-invasive function measurement requiring long restraint (>1h) without anesthesia, e.g., fMRI<br>- Invasive function measurement requiring long restraint (>1h) without anesthesia, e.g., electrophysiology<br>- Function measurement requiring head fixation<br>- Function measurements influencing glycemic levels causing hyper- or hypoglycemia, e.g., hyperglycemic glucose clamping, glucose tolerance test, insulin tolerance test, insulin clamp
**Severe**
- Function measurements requiring food/water deprivation within severe limits (see Table S9)

Table S12: Severity classification of pain tests.

**Mild**
Pain tests causing short-term mild pain at the pain detection threshold with possible slight tissue swelling including:<br>- Plantar/Hargraves test<br>- Von Frey<br>- Hot/cold plate<br>- Tail flick<br>- Tail immersion test
**Moderate**

- Pain tests causing short-term moderate pain, or chronic mild pain including:<br>o Nerve crush/ligation<br>o Localized subcutaneous injection of capsaicin<br>o Chemotherapy and toxin-induced neuropathy<br>- Pain tests requiring major surgery including:<br>o Colorectal distention requiring transmitter implantation through laparotomy (see Table S3)<br>- Pain test requiring footpad injection including<br>o Footpad injection with saline or pregnolone sulphate
**Severe**
Pain tests that cause short-term severe pain, or chronic moderate to severe pain including:<br>- Footpad injection with Complete Freund's Adjuvants, capsaicin or acrolein<br>- Writhing test

Table S13: Severity classification of genetically altered (GA) lines

	Sub-threshold (so GS2)	Mild	Moderate	Severe
**General**				
**Anatomy and appearance**				
**Macroscopically visible**	Visible anatomic variation (compared to wild type (WT) controls) but not affecting welfare, e.g., smaller animals, animal with shorter femur or snout, hyperpigmentation in zebrafish			GA lines causing jaundice<br>GA lines with anatomical abnormalities (in any body system) causing moribundity or mortality, e.g., Hunter disease
**Macroscopically not visible**	GA line normal on visual assessment but altered anatomy/physiology only detectable through histology/imaging or other tools, e.g., fluorescent GA lines, GA line with altered vasculature but clinically irrelevant			
	GA line normal on visual assessment but altered blood parameters only			

	detectable through blood analysis and not causing clinical disease, e.g., GA line with elevated cholesterol clinically irrelevant			
		**Growth and body weight**		
**Growth**	Genetic alteration causing smaller animals (compared to WT controls) not affecting welfare			Genetic alteration with severe growth retardation (runting)
**Body condition**	Normal condition (BCS3)	GA line causing overcondition (BCS4)	GA line causing obesity (BCS 5) or undercondition (BCS 2)	GA line causing wasting or emaciation (BCS1)
		**Lethality**		
**Mortality—in utero**	GA lines resulting in in utero lethality			
**Mortality—rodent pups up to 2 weeks**		GA lines resulting in death within 5 days post-partum (due to decreased perception of pain and distress) without other clinical signs	GA lines resulting in death between 5 days and 2 weeks post-partum (e.g., underdeveloped, leukopenia, anemia, microencephaly)	
**Mortality—rodents older than 2 weeks**		GA lines resulting in peracute death without other clinical signs		GA lines resulting in lethality (significantly earlier than WT strain) from 2 weeks post-partum on
**Mortality—zebrafish**		GA lines resulting in peracute death without other clinical signs		GA lines resulting in lethality
		**Pain**		
	GA lines resulting in hyperalgesia		GA lines resulting in long-term mild pain	GA lines resulting in long-term moderate pain or short-term severe pain
		**Behavior/emotional state**		

	Animals normal on visual assessment but altered behavior only detectable with behavioral or cognitive tests, e.g., GA lines of Alzheimer		GA lines resulting in long-term mild anxiety	GA lines resulting in long-term moderate anxiety or short-term severe anxiety
**Neoplasm**				
				GA lines resulting in tumor formation
**Per body system**				
**Neurology and sensory organs**				
**Sensory organs**	Phenotypes causing blindness and/or deafness in mice			
**Paresis and paralysis**	GA lines with slightly reduced strength/performance only detectable through specific tests like Rotarod or grip strength		GA lines resulting in moderate impairment of animal's motility with retained ability to reach food and water<br>Examples:<br>- Development of paresis like FUS-induced ALS<br>- Development of moderate arthritis	GA lines resulting in severe impairment of animal's motility and weight loss due to inability to reach food/water including:<br>- Development of paralysis like SOD ALS or Charcot-Marie-Tooth disease<br>- Development of severe arthritis
**Loss of coordination—gait**	GA lines resulting in altered gait only detectable through specific gait analysis tests	GA lines resulting in occasional (and visible) loss of coordination but animal can still move well, not affecting food and water intake	GA lines resulting in altered gait (e.g., hopping, wobbling, circling, wide stance and weak) but animal able to navigate the cage and able to eat and drink (food and water should	Marked abnormal gait impairing ability to move and function normally causing difficulties in eating and drinking

			be made accessible when necessary)	
**Seizures**		GA lines resulting in causing focal periodic seizures	GA lines resulting in occasional generalized seizures of short duration and normal afterwards	GA lines resulting in seizures of:<br>- Prolonged duration (minutes)<br>- Repetitive seizures affecting ability to maintain weight or behavior (hyperactivity or aggressive)<br>- W/o complete loss of consciousness during seizure<br>GA lines resulting in status epilepticus (seizure lasting > 5 min or recurrent epileptic seizures which can only be stopped by medication)<br>GA developing seizures causing mortality
**Respiratory system**				
				GA causing respiratory failure, e.g., GA lines of cystic fibrosis
**Heart and circulation**				
**Cardiomyopathy**		Transient and short-term intensified breathing after normal activity in the home cage; no permanent impairment of general condition	Global heart failure with permanent respiratory distress and impairment of the general condition, classification depends on expression of clinical signs (see Table S4)	

**Hypertension**	Slight hypertension up to 150 mmHg systolic blood pressure	GA lines resulting in hypertension up to 160 mmHg without impairment of the general condition and without strokes	GA lines resulting in short-term hypertension >180 mmHg systolic blood pressure with impairment of the general condition and with occurrence of spontaneous strokes	Slight hypertension up to 150 mmHg systolic blood pressure
**Coagulation defects**		GA lines with coagulation defects, severity depends on expression of clinical signs (see Table S4)		
**Endocrine system**				
				GA lines with diabetes like NOD mice, BB rats
**Gastro-intestinal tract**				
**IBD**		GA strains with inflammatory bowel disease (IBD), severity depends on expression of clinical signs (see Table S4)		
**Liver**				GA lines causing advanced liver disease with jaundice
**Urogenital**				
	GA lines resulting in sterility			
**Immune system**				
		GA lines with immunodeficiency		

Table S14: Severity classification of fetuses and premature animals

	Below threshold	Mild	Moderate	Severe

**In utero electroporation in mice**	In utero electroporation of mice born without a phenotype	In utero electroporation of mice born with a mild phenotype, e.g., causing mild stereotypic behavior	In utero electroporation of mice born with a moderate phenotype	In utero electroporation of mice born with a severe phenotype, e.g., causing severe seizures
**Fetal lamb surgery—spina bifida**	Lambs serving as control in spina bifida sheep model (not undergoing spina bifida creation)			Lambs born with spina bifida created surgically in utero
**Fetal lamb surgery—gastroschisis**	Surgical induction of gastroschisis in fetal lambs in utero. Lambs are killed before birth.			
**Fetal lamb surgery—others**	In utero blood collection in fetal lambs not affecting welfare after birth			
**Fetal rabbit surgery**	Fetal rabbits wherein diaphragmatic hernia was created but fetus is killed before birth	Rabbits born wherein growth retardation was caused in utero surgically (uteroplacental vessel ligation) or chemically		
**Norovirus infection in zebrafish**	Norovirus infection in zebrafish larvae 3 days post fertilization (dpf) not causing clinical signs beyond 120 hours post fertilization (hpf)			
